# Palateless Maxillary Implant‐Supported Overdentures: A Review of Patient Satisfaction and Clinical Outcomes Along With Case Series

**DOI:** 10.1002/ccr3.71516

**Published:** 2025-11-27

**Authors:** Zahra Jandaghian, Somayeh Zeighami

**Affiliations:** ^1^ Department of Prosthodontics, School of Dentistry Tehran University of Medical Sciences Tehran Iran; ^2^ Dental Research Center, Dentistry Research Institute and Department of Prosthodontics, School of Dentistry Tehran University of Medical Sciences Tehran Iran

**Keywords:** biomechanical phenomena, implant‐supported dental prosthesis, overdenture, patient satisfaction

## Abstract

Implant‐supported overdenture is a lifesaver treatment plan for edentulous patients. Implant numbers and positions in the maxilla and mandible are controversial, but in more studies on a minimum number of implants, which is at least four implants in the maxilla and two implants in the mandible, there is a consensus. Also, the attachment system (splinted vs. unsplinted) does not have a significant effect on bone loss, implant and prosthesis survival rate, and clinical complications. Palateless maxillary overdentures supported on four implants may improve taste sensation and reduce gag reflex without negatively affecting retention, resistance, and chewing ability. The aim of this study was to present three maxillary implant‐supported overdenture cases with four implants without palatal coverage.

## Key Clinical Message

Palateless maxillary implant‐supported overdentures retained by at least four implants can enhance patient comfort by improving taste sensation and reducing gag reflex, without compromising retention, stability, or chewing efficiency offering a clinically reliable and patient‐satisfying alternative to conventional full palate designs.

## 1 Introduction

Substitution of conventional complete dentures with implant‐supported overdentures has led to improved function, comfort, and patient satisfaction in comparison to conventional complete dentures [1]. There are no specific guidelines and comprehensive consensus regarding the optimum number and position of implants and the type of attachment system used in both arches [1]. In the mandibular overdenture, depending on different treatment plans, two to five implants in positions (A, B, C, D, and E) could be inserted [2]. Also, in maxillary overdenture, four to six implants without specifying the exact location of the implants have been considered [1]. It seems that the bone condition and the economic situation are the most determining factors in choosing the number and location of implants. Attachment systems (splinted vs. unsplinted) in both arches are usually selected considering vertical restorative space, inter‐implant distance, implant angulation, and residual ridge condition.

Studies about implant‐supported maxillary overdenture showed that four implants have similar results to six implants in terms of the survival rate of implants and overdenture and patient satisfaction [1], but at least four implants are recommended for more reliable clinical results [3]. Regarding implant‐supported mandibular overdenture, there is no consensus on the significant effect of the number of implants on marginal bone loss, implant survival rate, and clinical complications [4]. It seems that the type of attachment system (splinted vs. unsplinted) does not influence marginal bone loss, implant survival rate, and clinical complications significantly [5, 6, 7]. These implant‐supported overdentures provide more retention and stability and may require less need for full palatal coverage compared to conventional complete dentures. Numerous studies suggest that reducing palatal coverage may improve patient comfort, offering benefits such as enhanced taste sensation, better gag reflex control, and improved salivation, all while maintaining satisfactory retention [8]. This is particularly significant, as palatal coverage in conventional dentures has been associated with negative effects, including reduced taste sensation and impaired salivary flow [9].

Some research indicates that the retention of maxillary overdentures, whether conventional or implant‐supported, is predominantly influenced by tuberosity coverage, with less emphasis on palatal coverage [8]. The removal of palatal coverage in both conventional and implant‐supported overdentures has demonstrated minimal impact on retention, particularly when other areas, such as the tuberosities, are well‐covered [9]. Studies comparing the effects of palatal coverage on maxillary overdentures have shown that the absence of palatal support does not significantly affect retention or patient satisfaction, especially when implants or bars are utilized for support [10, 11].

This study aimed to present three maxillary implant‐supported overdenture cases with four implants and without palatal coverage, along with an evaluation of clinical outcomes. The patients gave written informed consent for the publication of their information and photos in this study.

## 2 Case History and Examination

### 2.1 Case 1

**FIGURE 1:**
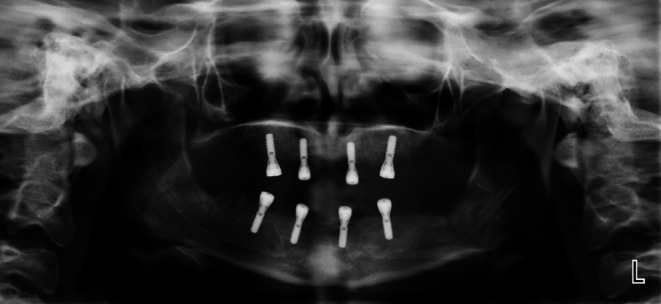
Panoramic view of Case 1.

**FIGURE 2:**
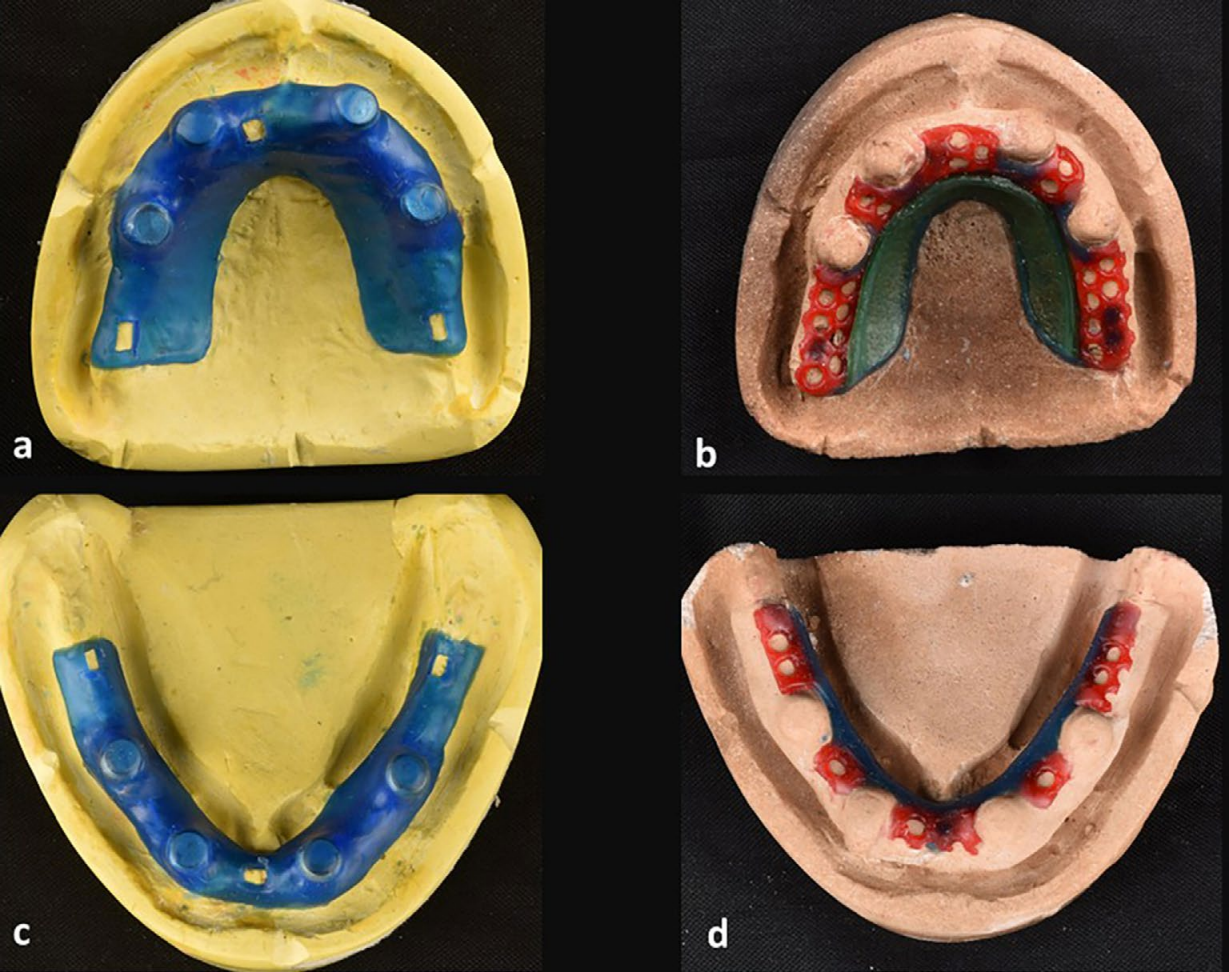
Relief and blackout and waxing the frameworks of Case 1. (a, b) maxilla. (c, d) mandible.

**FIGURE 3:**
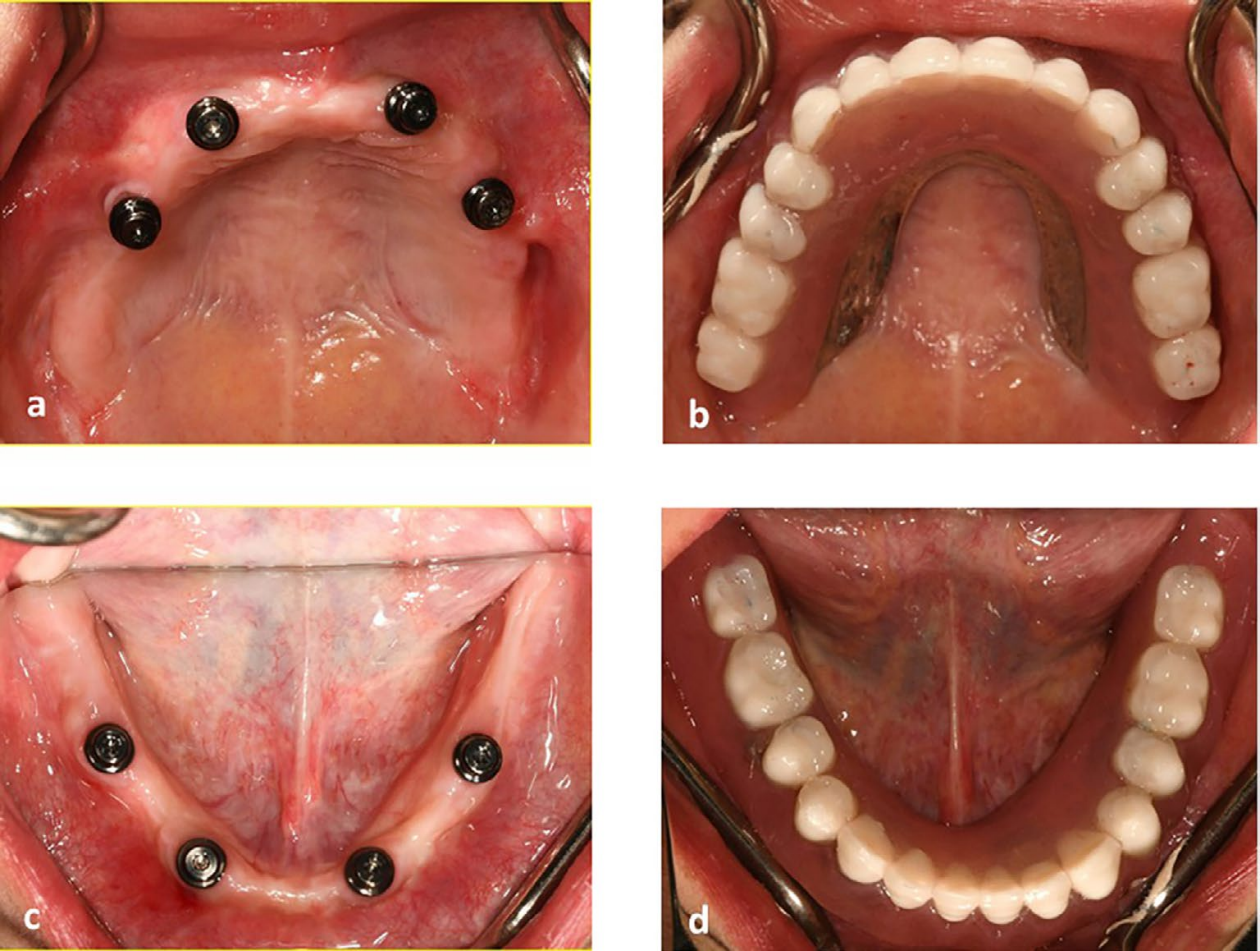
(a) Novaloc abutments in the maxilla. (b) maxillary palateless overdenture delivery. (c) Novaloc abutments in the mandible. (d) mandibular overdenture delivery.

A 21‐year‐old woman with hypoplastic amelogenesis imperfecta, who had undergone several dental procedures, including fillings and root canal treatments for the management of her condition, presented to the School of Dentistry, Tehran University of Medical Sciences, dissatisfied with her current dental condition and smile. A comprehensive dental history was taken, along with oral and radiographic evaluations. Based on the available evidence, most of her teeth were deemed hopeless and required extraction. During the consultation, the patient requested a full functional and esthetic reconstruction with implants at the lowest possible cost. CBCT evaluations confirmed that the quality and quantity of the bone were sufficient for implant placement without the need for additional surgical procedures, such as bone augmentation or sinus lift. Considering the patient's financial constraints and the need for increased lip support to reconstruct the smile line, the decision was made to proceed with maxillary and mandibular implant‐supported overdentures using four tissue‐level implants for each arch. Implant specifications are shown in Table 1. At the patient's request to enhance taste perception, a palate‐less maxillary overdenture was fabricated. Novaloc abutments (Straumann AG, Basel, Switzerland) were selected for each implant with proportionate gingival height, and bilateral balanced occlusion was established (Figures 1, 2, 3). After 6 months, the patient was asked to evaluate the overdentures from score 5 in terms of: taste sensation, reduced gag reflex, retention and stability, chewing efficacy, and patient satisfaction. The results for three patients are summarized in Table 2. Two‐year follow‐up revealed that the patient was still in post‐delivery condition.

**TABLE 1:** Implant specifications in three cases.

Case	Arch	Implants position^a^ ^,^ ^b^	Implants specification	Manufacturers
Case 1	Maxilla	#5, 7, 10, 12	Standard Plus Implants, SLActive, RN, 3.3 × 10	Straumann AG, Basel, Switzerland
	Mandible	A, B, D, E		
Case 2	Maxilla	#5, 7, 10, 12	SICace, 3.4 × 11.5	SIC invent AG, Basel, Switzerland
	Mandible	B, C, D		
Case 3	Maxilla	#3, 4, 9, 12, 13	Dio UFII Internal Submerged System, Regular Fixture (4 × 10) for all implants except #9 Narrow Fixture (3.3 × 11.5)	Dio IMPLANT, Busan, Republic of Korea
	Mandible	A, B, D, E	Dio UFII Internal Submerged System, Regular Fixture (4.5 × 10)	

^a^ Tooth numbering in the maxilla is according to the universal numbering system.

^b^ Implant area in mandible is according to Carl E. Misch, Dental Implant Prosthetics book.

**TABLE 2:** Evaluation of overdenture according to patient's survey (score out of 5).

Case	Taste sensation	Reduced gag reflex	Retention and stability	Chewing efficacy	Patient satisfaction
Case 1 (21‐years old woman)	5	5	5	4	5
Case 2 (27‐years old man)	5	5	4	4	5
Case 3 (63‐years old woman)	5	5	5	5	5

### 2.2 Case 2

**FIGURE 4:**
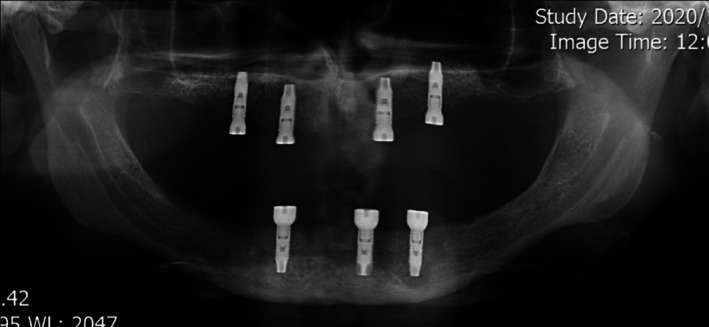
Panoramic view of Case 2.

**FIGURE 5:**
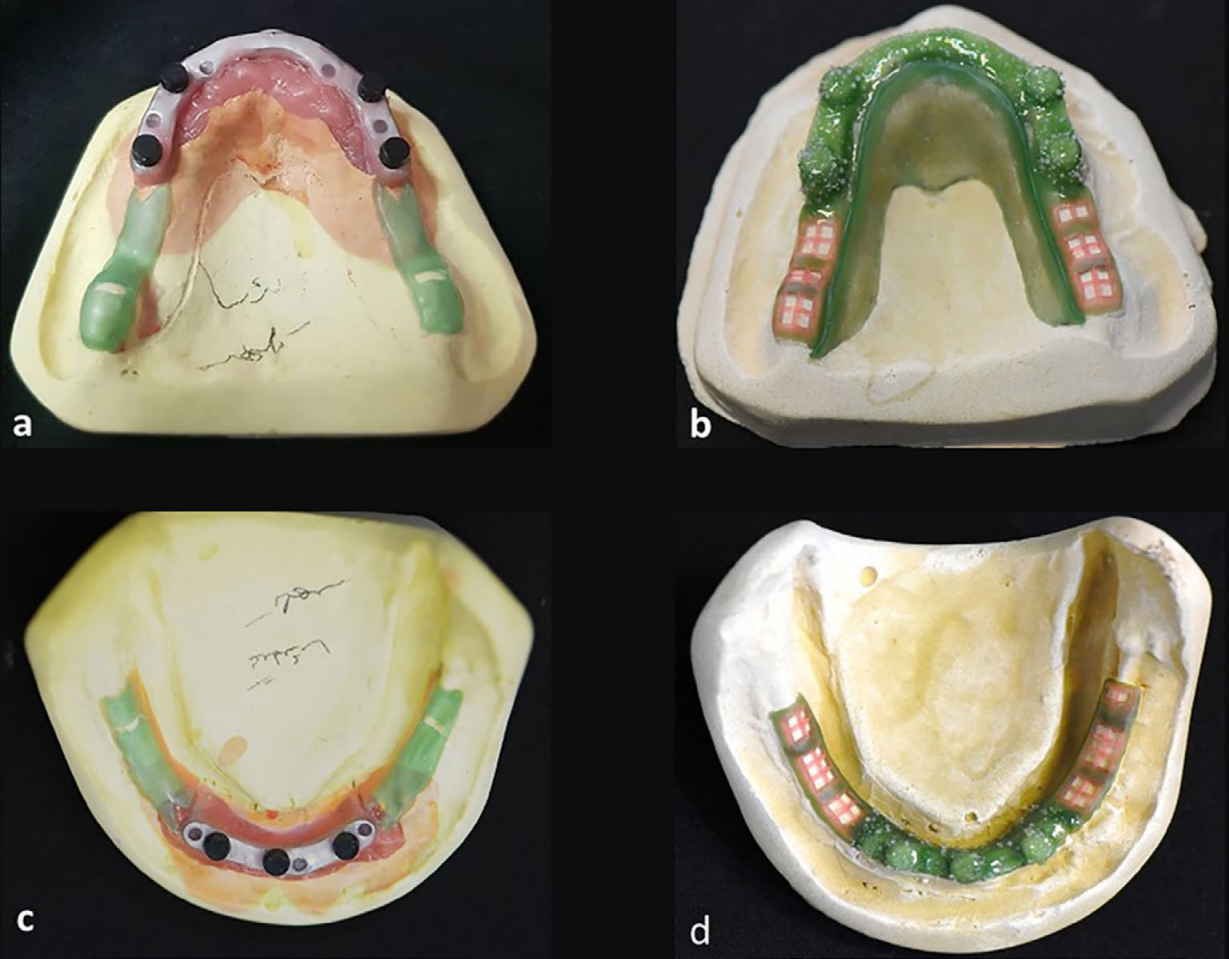
Relief and blackout and waxing the frameworks of Case 2. (a, b) maxilla. (c, d) mandible.

**FIGURE 6:**
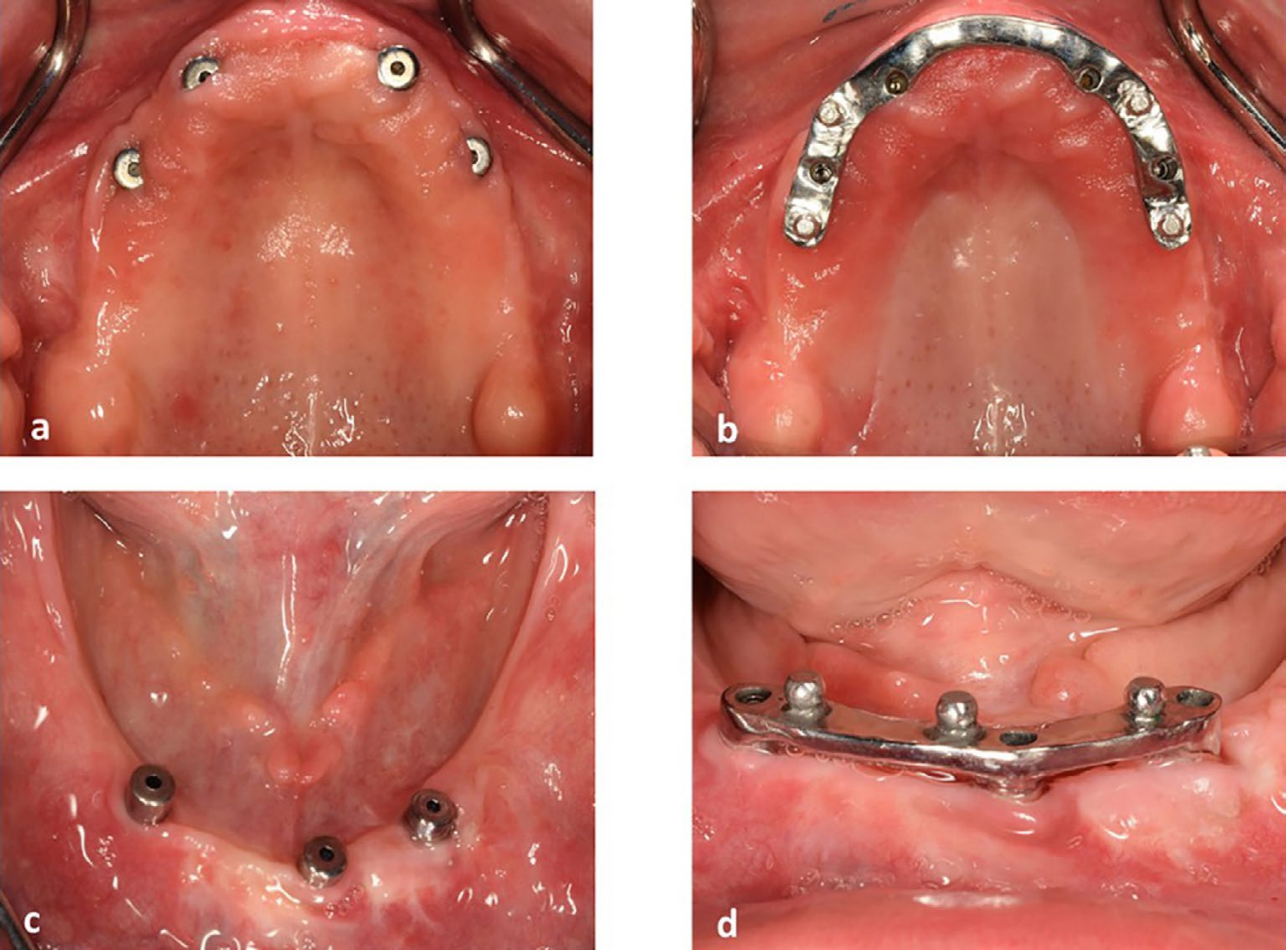
(a, b) Bar and ball attachment system in the maxilla. (c, d) bar and ball attachment system in the mandible.

**FIGURE 7:**
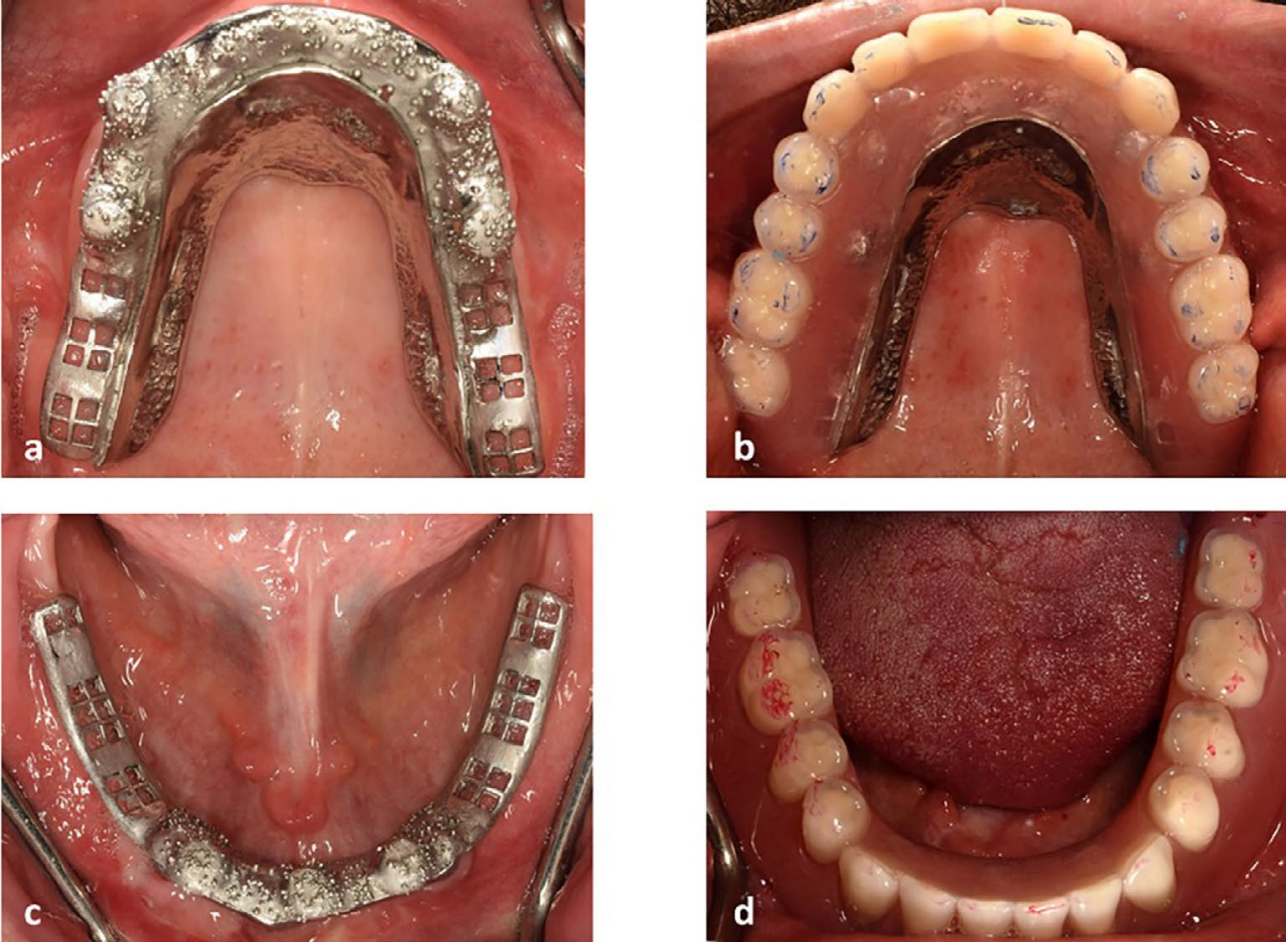
(a) Maxillary palateless framework try‐in. (b) maxillary palateless overdenture delivery. (c) mandibular framework try‐in. (d) mandibular overdenture delivery.

A 27‐year‐old man, with a history of generalized aggressive periodontitis that led to the extraction of all teeth, was referred to the School of Dentistry, Tehran University of Medical Sciences, for treatment of edentulism. A treatment plan involving maxillary and mandibular overdentures was proposed after intraoral and radiographic examinations. CBCT images were obtained using a radiographic guide, and surgery was performed with a nonlimiting surgical guide. Four bone‐level implants were placed in the maxilla, and three bone‐level implants were placed in the mandible. Implant specifications are shown in Table 1. The bar and ball attachment system was chosen for both arches. For this purpose, “Flex Star” universal cast‐to abutments for non‐precious alloy (SIC invent AG, Basel, Switzerland) and normal size castable ball attachments (RHEIN88, Bologna, Italy) were used. Then the casting procedure was accomplished. Based on the patient's request for improved taste sensation and reducing gag reflex, a palateless overdenture was fabricated for the maxilla, and bilateral balanced occlusion was established (Figures 4, 5, 6, 7). After 6 months, the patient was asked to evaluate the overdentures from score 5 in terms of: taste sensation, reduced gag reflex, retention and stability, chewing efficacy, and patient satisfaction. The results for three patients are summarized in Table 2. In a 4‐year follow‐up, the treatment was deemed successful, and the patient still reported high satisfaction.

**FIGURE 8:**
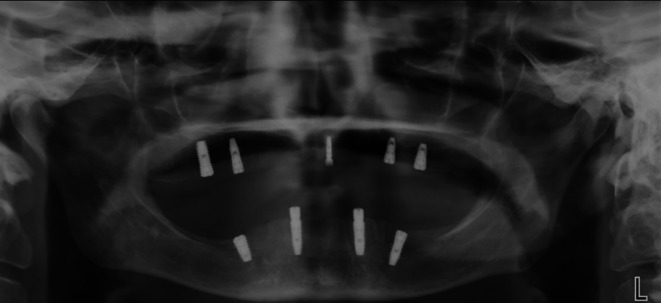
Panoramic view of Case 3.

**FIGURE 9:**
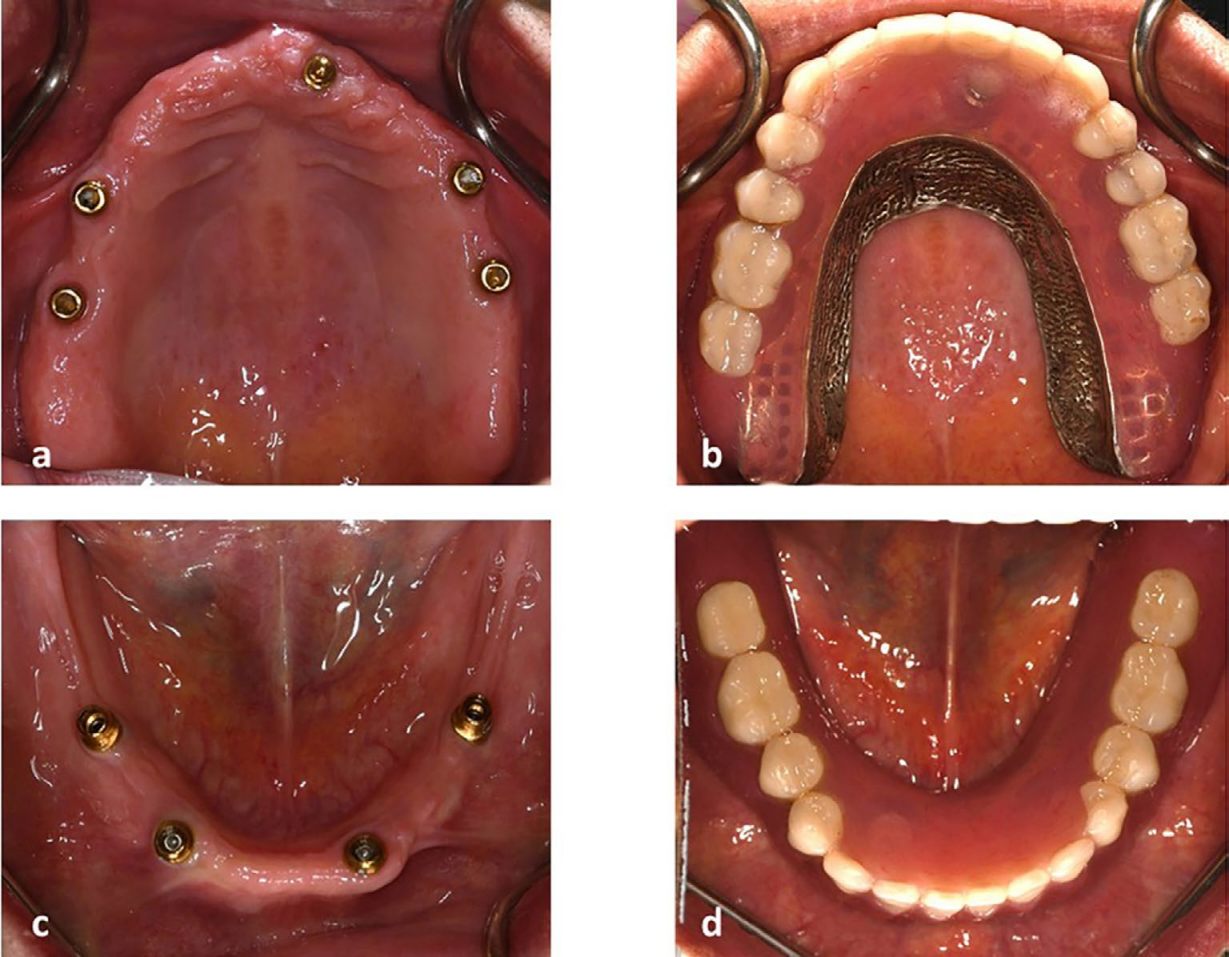
(a) Locator abutments in the maxilla. (b) maxillary palateless overdenture delivery. (c) Locator abutments in the mandible. (d) mandibular overdenture delivery.

### 2.3 Case 3

A 63‐year‐old edentulous woman with a history of diabetes mellitus presented to the School of Dentistry, Tehran University of Medical Sciences, seeking treatment for her edentulism. After a thorough assessment of intraoral and the quality and quantity of the residual bone in CBCT, a treatment plan involving implant‐supported overdentures was proposed. Four regular and one narrow bone‐level implants were placed in the maxilla, and four regular bone‐level implants were placed in the mandible. Implant specifications are shown in Table 1. Due to the patient's concerns about the gag reflex induced by the maxillary denture, and considering the presence of five implants in the maxilla, a palateless maxillary overdenture was recommended. Locator abutments (Dio IMPLANT, Busan, Republic of Korea) were selected except for the implant of site #9, which was chosen as the ball abutment (Dio IMPLANT, Busan, Republic of Korea), all with proportionate gingival height. Bilateral balanced occlusion was established (Figures 8 and 9). After 6 months, the patient was asked to evaluate the overdentures from score 5 in terms of: taste sensation, reduced gag reflex, retention and stability, chewing efficacy, and patient satisfaction. The results for three patients are summarized in Table 2. A 2‐year follow‐up showed that the treatment was still successful, along with patient satisfaction.

## 3 Discussion

The present study evaluated patient satisfaction with implant‐supported overdentures without palatal coverage. The results indicated a more favorable response, particularly in terms of reduced gag reflex and enhanced taste sensation, for this type of overdenture, regardless of the patient's age, sex, and the type of attachment system. Also, in the case of a palatal torus, this design is advantageous. These findings align with a previous study, which found that 80% of patients preferred overdentures with reduced palatal coverage due to greater comfort [12].

The frame design for this type of overdenture is an imitation of the horseshoe major connector of partial denture design. The medial borders of the frame should be positioned at the connection of the vertical and horizontal slopes of the palate. To enhance rigidity, the borders may be extended onto the horizontal surfaces of the palate to some extent. The frame should be symmetrical and have equal extension on both sides, with smooth and lightly curved borders. Like the other type of maxillary major connectors, a bead line for displacing adjacent soft tissue in the outer border of the horseshoe framework is required [13]. Lattice meshwork is attached to the framework on the edentulous ridge, positioned away from the attachments and their housings to facilitate future relining of these areas.

Also, lingualveolar sounds pronunciation such as d and t, which are created by contact of the tip of the tongue with the anterior area of the palate should be considered in forming the horseshoe framework and acrylic lingual area of anterior teeth [14].

This study confirms that the removal of palatal coverage did not negatively affect the stability of maxillary overdentures, which is consistent with previous research [9, 10, 11]. Proper occlusion was also found to contribute positively to the stability of palateless overdentures. Factors such as muscular forces, attachment systems, saliva, and implant positioning all play a role in retention, indicating that the presence of implants enhances retention regardless of the presence of palatal coverage [15].

Numerous studies have explored the occlusal scheme of implant‐supported overdentures. For each of the three patients in this study, a bilateral balanced occlusion was established based on the principles of complete denture occlusion. Khuder et al. examined different occlusal schemes in both conventional denture users and implant‐supported overdenture users, finding that the presence of implants improved chewing function. However, no significant difference was observed between various occlusal schemes in terms of mastication and patient satisfaction [16]. John Aarts et al. investigated two occlusal schemes, physiologic and lingualized, in patients using implant‐supported overdentures. Their findings showed that a larger number of patients preferred physiologic occlusion due to its better aesthetics and improved chewing ability, owing to the better penetration of cusped teeth into food. However, users of lingualized occlusion also reported benefits, including improved stability and retention of the denture [17]. Collectively, this body of research suggests that reducing palatal coverage in implant‐supported overdentures may enhance patient comfort without compromising retention, stability, or function. However, further clinical and biomechanical studies are needed to better understand the full implications of this design modification, particularly concerning marginal bone loss and implant survival rate. Thus, while promising, the use of palateless maxillary implant‐supported overdentures remains an area of ongoing research in prosthetic dentistry.

## 4 Conclusion

In conclusion, this case series suggests that implant‐supported maxillary overdentures with four implants and without palatal coverage can lead to patient satisfaction in terms of taste sensation, reduced gag reflex, retention and stability, and chewing ability regardless of the patient's age, sex, and the type of attachment system. If the patient has at least four implants in the maxillary arch, a palateless implant‐supported overdenture with splinted or unsplinted attachment systems could be a reasonable treatment option.

## Author Contributions

**Zahra Jandaghian:** data curation, project administration, writing – original draft, writing – review and editing. **Somayeh Zeighami:** conceptualization, data curation, project administration, supervision, writing – original draft, writing – review and editing.

## Funding

The authors have nothing to report.

## Conflicts of Interest

The authors declare no conflicts of interest.

## Data Availability

The data that support the findings of this study are available from the corresponding author upon reasonable request.

## References

[1] F. Di Francesco , G. De Marco , E. B. Capcha , et al., “Patient Satisfaction and Survival of Maxillary Overdentures Supported by Four or Six Splinted Implants: A Systematic Review With Meta‐Analysis,” BMC Oral Health 21, no. 1 (2021): 247, 10.1186/s12903-021-01572-6. 33962612 PMC8106178

[2] C. Misch , Dental Implant Prosthetics, 2nd ed. (Elsevier Mosby, 2015).

[3] F. Di Francesco , G. De Marco , U. A. Gironi Carnevale , M. Lanza , and A. Lanza , “The Number of Implants Required to Support a Maxillary Overdenture: A Systematic Review and Meta‐Analysis,” Journal of Prosthodontic Research 63, no. 1 (2019): 15–24, 10.1016/j.jpor.2018.08.006. 30269880

[4] M. Roccuzzo , F. Bonino , L. Gaudioso , M. Zwahlen , and H. J. Meijer , “What Is the Optimal Number of Implants for Removable Reconstructions? A Systematic Review on Implant‐Supported Overdentures,” Clinical Oral Implants Research 23, no. Suppl 6 (2012): 229–237, 10.1111/j.1600-0501.2012.02544.x. 23062145

[5] K. Ahmed , “Splinted Versus Unsplinted Overdenture Attachment Systems—No Difference in Clinical Outcomes,” Evidence‐Based Dentistry 20, no. 1 (2019): 28–29, 10.1038/s41432-019-0006-9. 30903127

[6] S. Prasad , L. P. Faverani , J. F. Santiago Junior , C. Sukotjo , and J. C. Yuan , “Attachment Systems for Mandibular Implant‐Supported Overdentures: A Systematic Review and Meta‐Analysis of Randomized Controlled Trials,” Journal of Prosthetic Dentistry 132, no. 2 (2024): 354–368, 10.1016/j.prosdent.2022.08.004. 36115712

[7] R. S. Leão , S. L. D. Moraes , B. C. E. Vasconcelos , C. A. A. Lemos , and E. P. Pellizzer , “Splinted and Unsplinted Overdenture Attachment Systems: A Systematic Review and Meta‐Analysis,” Journal of Oral Rehabilitation 45, no. 8 (2018): 647–656, 10.1111/joor.12651. 29761853

[8] A. Zembic , A. Tahmaseb , and D. Wismeijer , “Within‐Subject Comparison of Maxillary Implant‐Supported Overdentures With and Without Palatal Coverage,” Clinical Implant Dentistry and Related Research 17, no. 3 (2015): 570–579, 10.1111/cid.12125. 23899103

[9] K. T. Ochiai , B. H. Williams , S. Hojo , R. Nishimura , and A. A. Caputo , “Photoelastic Analysis of the Effect of Palatal Support on Various Implant‐Supported Overdenture Designs,” Journal of Prosthetic Dentistry 91, no. 5 (2004): 421–427, 10.1016/s0022391304000927. 15153848

[10] T. Takahashi , T. Gonda , Y. Mizuno , Y. Fujinami , and Y. Maeda , “Influence of Palatal Coverage and Implant Distribution on Implant Strain in Maxillary Implant Overdentures,” International Journal of Oral & Maxillofacial Implants 31, no. 5 (2016): e136–e142, 10.11607/jomi.4535. 27632280

[11] T. Takahashi , T. Gonda , A. Tomita , and Y. Maeda , “Effect of Attachment Type on Implant Strain in Maxillary Implant Overdentures: Comparison of Ball, Locator, and Magnet Attachments. Part 2: Palateless Dentures,” International Journal of Oral & Maxillofacial Implants 33, no. 2 (2018): 357–364, 10.11607/jomi.6157. 29534124

[12] J. Balaguer , B. García , M. Peñarrocha , and M. Peñarrocha , “Satisfaction of Patients Fitted With Implant‐Retained Overdentures,” Medicina Oral, Patología Oral y Cirugía Bucal 16, no. 2 (2011): e204–e209. 20711152

[13] R. D. Phoenix , D. R. Cagna , and C. F. DeFreest , Stewart's Clinical Removable Partial Prosthodontics, 4th ed. (Quintessence Publishing Co Inc, 2008).

[14] G. Zarb , J. Hobkirk , S. Eckert , and R. Jacob , Prosthodontic Treatment for Edentulous Patients, 13th ed. (Mosby, 2013).

[15] O. Fromentin , C. Lassauzay , S. Abi Nader , J. Feine , and R. F. de Albuquerque Junior , “Testing the Retention of Attachments for Implant Overdentures—Validation of an Original Force Measurement System,” Journal of Oral Rehabilitation 37, no. 1 (2010): 54–62, 10.1111/j.1365-2842.2009.02020.x. 19912482

[16] T. Khuder , N. Yunus , E. Sulaiman , N. Ibrahim , T. Khalid , and M. Masood , “Association Between Occlusal Force Distribution in Implant Overdenture Prostheses and Residual Ridge Resorption,” Journal of Oral Rehabilitation 44, no. 5 (2017): 398–404, 10.1111/joor.12504. 28295492

[17] J. M. Aarts , A. G. Payne , and W. M. Thomson , “Patients' Evaluation of Two Occlusal Schemes for Implant Overdentures,” Clinical Implant Dentistry and Related Research 10, no. 3 (2008): 140–156, 10.1111/j.1708-8208.2007.00070.x. 18218055

